# Dynamic Changes in Microbial Composition During Necrotizing Soft-Tissue Infections in ICU Patients

**DOI:** 10.3389/fmed.2020.609497

**Published:** 2021-03-04

**Authors:** Michael Thy, Sébastien Tanaka, Alexy Tran-Dinh, Lara Ribeiro, Brice Lortat-Jacob, Julia Donadio, Nathalie Zappella, Mouna Ben-Rehouma, Parvine Tashk, Aurelie Snauwaert, Enora Atchade, Nathalie Grall, Philippe Montravers

**Affiliations:** ^1^Assistance Publique - Hôpitaux de Paris (AP-HP), Department of Anesthesiology and Critical Care Medicine, Bichat-Claude Bernard Hospital, Paris, France; ^2^EA 7323 - Pharmacology and Therapeutic Evaluation in Children and Pregnant Women, Paris Descartes University, Sorbonne Paris Cité University, Paris, France; ^3^Réunion Island University, French Institute of Health and Medical Research (INSERM), U1188 Diabetes atherothrombosis Réunion Indian Ocean (DéTROI), CYROI Plateform, Saint-Denis de La Réunion, France; ^4^Université de Paris, UFR Denis Diderot, Paris, France; ^5^French Institute of Health and Medical Research (INSERM) U1148, Laboratory for Vascular Translational Science, Paris, France; ^6^Assistance Publique - Hôpitaux de Paris (AP-HP), Department of General and Visceral Surgery, Bichat-Claude Bernard Hospital, Paris, France; ^7^Assistance Publique - Hôpitaux de Paris (AP-HP), Department of Orthopedic Surgery, Bichat-Claude Bernard Hospital, Paris, France; ^8^Paris-Saclay University, French Institute of Health and Medical Research, INSERM UMR 1195, Le Kremlin-Bicêtre, France; ^9^Assistance Publique - Hôpitaux de Paris (AP-HP), Department of Bacteriology, Bichat-Claude Bernard Hospital, Paris, France; ^10^French Institute of Health and Medical Research (INSERM), IAME, UMR 1137, Paris, France; ^11^French Institute of Health and Medical Research (INSERM) U1152, Physiopathology and Epidemiology of Respiratory Diseases, Paris, France

**Keywords:** necrotizing soft-tissue infections (NSTI), intensive care unit (ICU), sepsis, multidrug-resistant (MDR) bacteria, outcome, antimicrobial therapy

## Abstract

**Introduction:** Recent studies described the threat of emerging multidrug-resistant (MDR) bacteria in intensive care unit (ICU) patients, but few data are available for necrotizing skin and soft tissue infections (NSTI). In a cohort of ICU patients admitted for NSTI, we describe the dynamic changes of microbial population during repeated surgeries.

**Materials and Methods:** This retrospective study compiled consecutive cases admitted for the management of severe NSTI. Clinical characteristics, NSTI features, morbidity and mortality data were collected. The microbiological characteristics of surgical samples obtained during initial surgery were compared with those obtained during the first reoperation, including persistence of initial pathogens and/or emergence of microorganisms. Risk factors for emergence of microorganisms and MDR bacteria were assessed by univariable and multivariable analyses.

**Results:** Among 100 patients {63% male, 58 years old [interquartile ratio (IQR) 50–68]} admitted for NSTI, 54 underwent reoperation with a median [IQR] delay of 3 (1–7) days. Decreased proportions of susceptible strains and emergence of Gram-negative bacteria, including *Pseudomonas aeruginosa*, staphylococci and enterococci strains, were reported based on the cultures of surgical specimen collected on reoperation. On reoperation, 22 (27%) of the isolated strains were MDR (*p* < 0.0001 vs. MDR bacteria cultured from the first samples). Broad-spectrum antibiotic therapy as first-line therapy was significantly associated with a decreased emergence of microorganisms. Adequate antibiotic therapy from the initial surgery did not modify the frequency of emergence of microorganisms (*p* = 0.79) and MDR bacteria (*p* = 1.0) or the 1-year survival rate.

**Conclusion:** The emergence of microorganisms, including MDR bacteria, is frequently noted in NSTI without affecting mortality.

## Introduction

Necrotizing soft-tissue infections (NSTI) require early diagnosis, adequate early surgical source control and appropriate antibiotic management according to recent international recommendations (1, 2). However, despite early management of NSTI, morbidity and mortality remain high (3). Recent reports underlined the emergence of multidrug resistant (MDR) bacteria in the ICU (4, 5), but few data focused on NSTI (6). Risk stratification for antibiotic resistance in skin and soft tissue infections remains poorly investigated, and few specific risk factors have been identified for specific bacterial species or MDR pathogens (7). Repeated inspection and additional debridement are recommended, which could help to re-assess antibiotic therapy through additional microbiological samples (1, 8–13). Many patients require repeated procedures and prolonged anti-infective treatments that might increase the risk of emergence of MDR bacteria. The timeline of the changes of such pathogens has been minimally addressed in NSTI. A better understanding of dynamic changes of microbial population in ICU patients managed for NSTIs could be helpful for optimizing anti-infective therapy.

The goals of the present study were to describe an ICU cohort of patients admitted for NSTI, define the dynamic changes of microbial population during repeated surgeries, and assess the risk factors for emergence of MDR bacteria and the prognosis of these microbiological issues.

## Materials and Methods

### Study Population

This retrospective study compiled all consecutive patients admitted to our University Hospital ICU for the management of NSTI from April 2009 to March 2019. The identification of the cases was made through the database of the health information system of our hospital. The ICD-10 scores were used, initially including a search of all skin and subcutaneous lesions reported in patients admitted to the ICU. Then, the analysis of the medical reports allowed to select those with a diagnosis of NSTI. Patients with uncertain diagnosis or without NSTI were excluded.

The retrospective nature of our study waived the need for signed informed consent. This study was declared to the French Data Protection Authority (CNIL: 2096382v0) and was approved by the French Institutional Review Board (Comité d'Éthique de la Recherche en Anesthésie-Réanimation, IRB number 00010254-2020-153).

### Surgical Procedures

The index surgical exploration was performed to confirm the diagnosis and to achieve source control through aggressive debridement of infected necrotic tissue. Surgical samples were obtained for microbiological analysis. Wound care, including cleaning and trimming, was performed every 24 or 48 h at bedside according to the local aspect. In case of unfavorable systemic and/or local evolution, a second surgical exploration was performed in the operating theater to ensure the adequacy of source control and to collect additional microbiological samples (2, 14). Only the first two procedures were analyzed (only two patients had subsequent samples).

### Microbiological Data

Blood cultures were collected at the time of ICU admission and repeated in clinical situations evocative of sepsis or bacteraemia. Fine needle aspiration specimens were taken at the time of admission before the source control procedure. Direct needle aspiration was focused on the leading edge, mid-lesion or bullae according to conventional recommendations (15, 16). Peroperative specimens were obtained from deep tissues collected during initial surgical source control or at the time of reoperation (17, 18). Swabs and samples from non-sterile sites (such as open bullaes) were not considered. Cases of NSTI based on inconsistent samples (swabs, non-sterile sites, open skin injury, etc.) were excluded.

Microbiological samples were immediately sent to the laboratory for bacterial and fungal cultures. Samples were processed according to the laboratory standard methods. Plates were incubated for 48 h at 35°C. All morphologically distinct colonies were identified by standard bacteriologic techniques and tested for antibiotic susceptibility by the disk diffusion method according to EUCAST^1^. The carbapenem resistant Gram-negative bacilli strains were screened for *bla*_KPC_, *bla*_VIM_, *bla*_IMP_, *bla*_NDM_, and *bla*_OXA−48_ genes by home-made PCR then using the Cepheid Xpert® Carba-R assay (Cepheid, Sunnyvale, USA).

Results of microbiological cultures of samples collected during first and second operations were compared in terms of species and susceptibility profile. A persisting micro-organism was defined as the same microorganism (bacteria or fungi) isolated from one sample to the next on the basis of identification and susceptibility profile without analysis of genetic relatedness (19). Emerging microorganisms were defined as microorganisms (bacteria or fungi) isolated from the sample of the second operation and not from the first one on the basis of identification and/or susceptibility profile (19). MDR and extensively drug resistant (XDR) bacteria were defined according to international definitions (20).

### Empirical Therapy

Empirical anti-infective therapy (EAT) taking into account clinical severity was systematically initiated on the first operation and targeted Gram-positive and Gram-negative aerobic and anaerobic microorganisms. The conventional regimen was based on beta-lactams with anti-anaerobic activity (amoxicillin-clavulanate, piperacillin/tazobactam or imipenem/cilastatin) or third-generation cephalosporin associated with metronidazole combined ± aminoglycosides and ± anti-Gram-positive agents in case of suspicion of resistant bacteria (2, 21). Documented anti-infective therapy was adapted to the results of identification and susceptibility testing (≥48 h) and was defined as adequate when targeting all the cultured microorganisms. The same rule was applied to the first reoperation.

### Data Collection

Demographic data and severity (SAPS-II and SOFA) scores were recorded on ICU admission (22, 23). The severity of the underlying medical condition [malignancy, obesity, diabetes mellitus, vascular disease, alcohol use, active smoking, and immunosuppression (immune deficiency, HIV, or chronic corticosteroids or antineoplastic medication)] was assessed. The Charlson comorbidity index and the Laboratory Risk Indicator for Necrotizing Fasciitis score (LRINEC) were calculated (24, 25). Characteristics of the NSTI were assessed [site (cephalic, trunk, pelvis, and limbs), multiple locations, amputation, and stoma]. The proportion of injured skin surface was assessed according to the nine rules for burns (26). This estimate was calculated independently by two authors (MT and ST) based on the anatomic descriptions of the operative reports and the pictures taken by the surgeons and intensivists. Clinical and therapeutic features were recorded on admission and during the ICU stay, including sepsis shock, renal failure, and need for mechanical ventilation, vasoactive support, renal replacement therapy and reoperation. The length of ICU and hospital stay and the mortality rates at day 28, day 90, and 1 year after admission were collected.

### Statistical Analysis

Results are expressed in medians and IQR as well as absolute numbers and proportions for categorical data. Continuous data were compared using the Mann-Whitney U test. Categorical data were analyzed using the Chi2 or Fisher exact test. All reported statistical tests were 2-sided, and *p* values <0.05 were considered significant.

Risk factors for emerging microorganisms and emerging MDR bacteria were analyzed in univariable analysis with univariable logistic regression or with non-parametric tests corresponding to unpaired Wilcoxon test for continuous variables and Fisher's exact test for discrete variables when univariable logistic models did not converge (27, 28). Survival curves were estimated by the Kaplan–Meier method, and differences in survival between groups were assessed by the log-rank test. For multivariable analyses, a logistic regression model was used due to a high number of predictors compared with the number of events/non-events (29). Odds ratios (ORs) and 95% confidence intervals (CIs) were calculated. For comparison, we have reported results from normal univariable and adjusted logistic regression. Variables with a *p*-value <0.1 in univariable analysis were entered in a multivariable logistic regression analysis with stepwise selection. The logistic regression models analyzed separately the variables available on admission and those collected during ICU stay. Statistical analyses were performed using the R software version 3.6.2.

## Results

### Study Population

Overall, 100 consecutive patients [63% men, 58 (IQR 50–68) years old] were admitted to our ICU for the diagnosis of NSTI with a median delay of 2 (0–5) days between hospital admission and index surgery. The general characteristics of the patients are presented in Table 1. The median body surface area of the lesions was 4.5% (4.5–9), and limbs, pelvis, cephalic and trunk areas were involved in 52, 28, 23, and 13% of the patients, respectively. Multiple locations were observed in 14% of the patients.

**Table 1:** Characteristics and comparison of patients who underwent one surgical procedure with the patients who underwent two surgical procedures.

***n* = 100**	**Overall**	**One surgical procedure**	**Two surgical procedures**	
**Variables**	**100**	**46**	**54**	***p***
**Demographic data**				
Age (years), median (IQR)	58 (50–68)	62 (55–69)	56 (48–63)	0.016
Male gender, *n* (%)	63 (63)	27 (59)	36 (67)	0.539
Weight (kg), median (IQR)	82 (67–95)	85 (69–101)	80 (66–92)	0.309
BMI (kg/m^2^), median (IQR)	28 (23–34)	29 (23–37)	27 (22–31)	0.194
**Underlying diseases**				
Chronic obstructive pulmonary disease, *n* (%)	15 (15)	6 (13)	9 (17)	0.822
Cardiovascular disease, *n* (%)	34 (34)	12 (27)	22 (41)	0.238
Diabetes mellitus, type I or II, *n* (%)	40 (40)	18 (39)	22 (41)	1.000
Peripheral vascular disease, *n* (%)	17 (17)	9 (20)	8 (15)	0.716
Immunosuppression, *n* (%)	10 (10)	5 (11)	5 (9)	1.000
Cancer, *n* (%)	23 (23)	11 (24)	12 (22)	1.000
Active smoking	54 (54)	27 (59)	27 (50)	0.504
Alcohol use, *n* (%)	20 (20)	10 (22)	10 (19)	0.880
Use of steroids or NSAI drugs, *n* (%)	7 (7)	2 (40)	5 (42)	1.000
Charlson score median (IQR)	4 (3–7)	3 (2–5)	3 (2–7)	0.943
**CT scan on the first examination**, ***n*** **(%)**	49 (49)	24 (52)	25 (49)	0.915
Median delay from first exam to CT scan, days (IQR)	1 (0–4)	2 (0–4)	1 (0–4)	0.981
Deep abscess, *n* (%)	31 (31)	10 (39)	21 (60)	0.160
Bullae, *n* (%)	27 (27)	11 (42)	16 (46)	0.997
Fasciitis signs, *n* (%)	21 (21)	9 (35)	12 (34)	1.000
Osteitis signs, *n* (%)	6 (6)	3 (12)	3 (9)	1.000
**Severity criteria**				
SAPS II score on admission, median (IQR)	28 (23–37)	34 (21–52)	37 (27–46)	0.547
SOFA score on admission, median (IQR)	5 (3–6)	4 (3–6)	5 (4–7)	0.161
LRINEC score on admission, median (IQR)	2 (1–4)	2 (1–4)	2 (1–5)	0.552
**Treatment**				
Vaso-active support on admission, *n* (%)	53 (53)	18 (41)	35 (65)	0.031
Renal replacement therapy, *n* (%)	22 (22)	6 (14)	16 (31)	0.081
Length of mechanical ventilation, median (IQR)	3 (0–13)	1 (0–7)	9 (1–15)	0.018
Antibiotic duration, median (IQR)	15 (11–18)	13 (7–15)	16 (15–28)	0.001
Vacuum-assisted closure device, *n* (%)	28 (28)	11 (24)	17 (32)	0.504
Skin graft, *n* (%)	24 (24)	6 (13)	18 (33)	0.020
Amputation, *n* (%)	13 (13)	7 (15)	6 (11)	0.566
**Outcomes**				
Length of hospital stay in days, median (IQR)	40 (18–59)	35 (9–58)	43 (27–57)	0.112
Length of ICU stay in days, median (IQR)	7 (2–19)	3 (1–9)	11 (6–27)	<0.001
Death, *n* (%)	25 (25)	10 (22)	15 (28)	0.644

Initial surgery was always performed under general anesthesia with intubation and mechanical ventilation. The median duration of mechanical ventilation was 3 days [0–13]. All patients received EAT at the time of surgery, mainly with piperacillin + tazobactam (*n* = 33), cephalosporin (*n* = 21), amoxicillin-clavulanic acid (*n* = 25), aminoglycoside (*n* = 24), carbapenem (*n* = 18) and vancomycin (*n* = 7). EAT with antitoxin activity was used in 40% of the cases [clindamycin (*n* = 26) and linezolid (*n* = 12)]. EAT was not adequate for 12 patients (12%). Patients with *Enterobacterales* isolated from surgical samples were more severe than other patients with an increased frequency of vasoactive support (71 vs. 43% of patients with other microorganisms, *p* = 0.018) and renal replacement therapy (38 vs. 16%, *p* = 0.032).

A second procedure was required in 54 patients with a median delay of 3 (1–7) days after index surgery. No significant difference was found between the patients who underwent one single surgical procedure and patients who underwent at least a second operation except age (*p* = 0.016) and vasoactive support on admission (*p* = 0.031) (Table 1). The main indications for re-interventions were the persistence of necrosis in the lesions, worsening of local or systemic clinical signs, and worsening of biological parameters. At the time of this second procedure, the median SOFA score was 4 (2–8) with a median SOFA score difference between the index surgery and the day of reoperation of 0 [−3; +3] and a median hyperleukocytosis of 15 (10–23) G/L with a median difference in leucocytosis of 1 (−2; +5) G/L.

### Microbiological Findings Over Time

The cultures of surgical samples collected on the initial surgery yielded 174 microorganisms (168 bacteria and 6 fungi) (Table 2). The predominant pathogens were Gram-positive bacteria [*n* = 87 (50%)] with a majority of streptococci [*n* = 45 (27%)]. During the initial procedure, 9 (5%) MDR strains were isolated in the samples of 7 (7%) patients (Table 2). The microbial combinations obtained during the first surgery are presented in Supplementary Table 1. Bacteraemias were reported in 24 patients and mostly related to Gram-positive cocci.

**Table 2:** Microorganisms isolated from the first and second surgery.

	**First surgery**	**Reoperation**	**Emerging organisms**	**Persisting organisms**
Aerobes, *n* (%)	146 (84)	84 (88)	56	28
Gram-positive bacteria, *n* (%)	87 (50)	37 (39)^*^	20	17
Enterococci, *n* (%)	16 (9)	7 (7)	4	3
*E. faecalis, n* (%)	12 (7)	3 (4)	2	1
*E. faecium, n* (%)	1 (1)	3 (4)	2	1
Streptococci, *n* (%)	45 (27)	10 (12)^*^	2	8
GAS, *n* (%)	12 (7)	1 (1)	0	1
Staphylococci, *n* (%)	26 (15)	20 (21)	14	6
*Staphylococcus aureus, n* (%)	15 (9)	8 (10)	6	2
Coagulase-negative staphylococci, *n* (%)	11 (7)	10 (12)	8	2
Gram-negative bacteria, *n* (%)	59 (35)	47 (52)^*^	36	11
*Enterobacterales, n* (%)	48 (28)	33 (35)	26	17
*Escherichia coli, n* (%)	21 (12)	15 (18)	10	5
*Enterobacter spp., n* (%)	2 (1)	6 (7)^*^	6	0
*Klebsiella spp., n* (%)	9 (5)	12 (15)^*^	10	2
Non-fermenting Gram-negative bacilli, *n* (%)	11 (7)	14 (17)^*^	10	4
*Pseudomonas aeruginosa, n* (%)	9 (5)	14 (17)^*^	10	4
Anaerobes, *n* (%)	22 (13)	5 (6)	3	2
*Bacteroides* spp., *n* (%)	9 (5)	4 (5)	3	1
Fungi, *n* (%)	6 (4)	6 (7)	6	0
*Candida albicans, n* (%)	1 (1)	2 (2)	2	0
Total number of strains, *n* (%)	174 (100)	95 (100)	65	30
Total number of MDR bacteria, *n* (%)	9 (5)	22 (27)^*^	19	3
MRSA	2	3	3	0
Overproduction of intrinsic or plasmid-encoded AmpC	2	5^*^	3	2
ESBL producing *Enterobacterales*	4	9^*^	8	1
Carbapenemase producing *Enterobacterales*	1	2	2	0
XDR *Pseudomonas aeruginosa*	0	3^*^	3	0

*GAS, group A streptococci; MRSA, methicillin-resistant Staphylococcus aureus; AmpC, cephalosporinase; ESBL, extended spectrum betalactamase; XDR, extensively drug resistant*.

The variables followed by an asterisk * indicate those with a significant difference between 1st surgery and re-intervention among patients.

The second surgical procedure performed in 54 (54%) patients led to the isolation of 95 microorganisms (89 bacteria and 6 fungi) (Table 2). The predominant pathogens were Gram-negative bacteria [*n* = 47 (52%)] with a majority of *Enterobacterales* [*n* = 33 (35%)] and *Pseudomonas aeruginosa* [*n* = 14 (17%)]. On reoperation, 22 (27%) of the isolated strains were MDR (*p* < 0.0001 vs. MDR bacteria cultured from the first samples). The emergence of two carbapenemase-producing bacteria (*Klebsiella pneumoniae* NDM) was reported during an outbreak in our ICU. Proportions of microorganisms, MDR bacteria and microbiological comparisons between the two surgical procedures are displayed in Table 2. The incidence of enterococci, *E. coli*, and non-fermenting Gram-negative bacilli were significantly different based on the NSTI location (Supplementary Table 2). The highest proportions of anaerobes were cultured during the first surgery for pelvic NSTIs reaching 20% of the isolates, while the highest proportions of Gram-positive bacteria were observed in cephalic NSTIs (65% of the isolates).

### Persisting Microorganisms

Among the microorganisms isolated during the first surgery, 30 (18%) were persistent on reoperation (Table 2), including *P. aeruginosa* [*n* = 4 (10%)] and *Enterobacterales* [*n* = 17 (43%)]. Lower limbs and pelvic region were the two main sites in which persisting bacteria were identified. No clinical characteristics or co-morbidities were significantly associated with persistence of microorganisms.

In univariable analysis, a significant association was observed between persisting microorganisms and a post-operative NSTI; the surface of altered skin; the SAPS II score on admission; the need for renal replacement therapy; the number of nosocomial infections, especially hospital-acquired pneumonia; the polymicrobial nature of initial infection; the presence of anaerobes, *Enterobacterales* or *P. aeruginosa* in the first surgical samples. Adequacy of EAT, its duration and its delay of initiation were not associated with persisting microorganisms. In multivariable analysis, the nosocomial context of NSTI [OR 4.4 (95% CI 1.4–14.2), *p* = 0.01], the presence of hospital-acquired pneumonia [OR 7.8 (95% CI 1.4–67), *p* = 0.03] and the polymicrobial nature of the initial infection [OR 3.8 (95% CI 1.2–13.5), *p* = 0.03] were independent factors for persisting microorganisms on reoperation.

### Emerging Microorganisms

**Figure 1:**
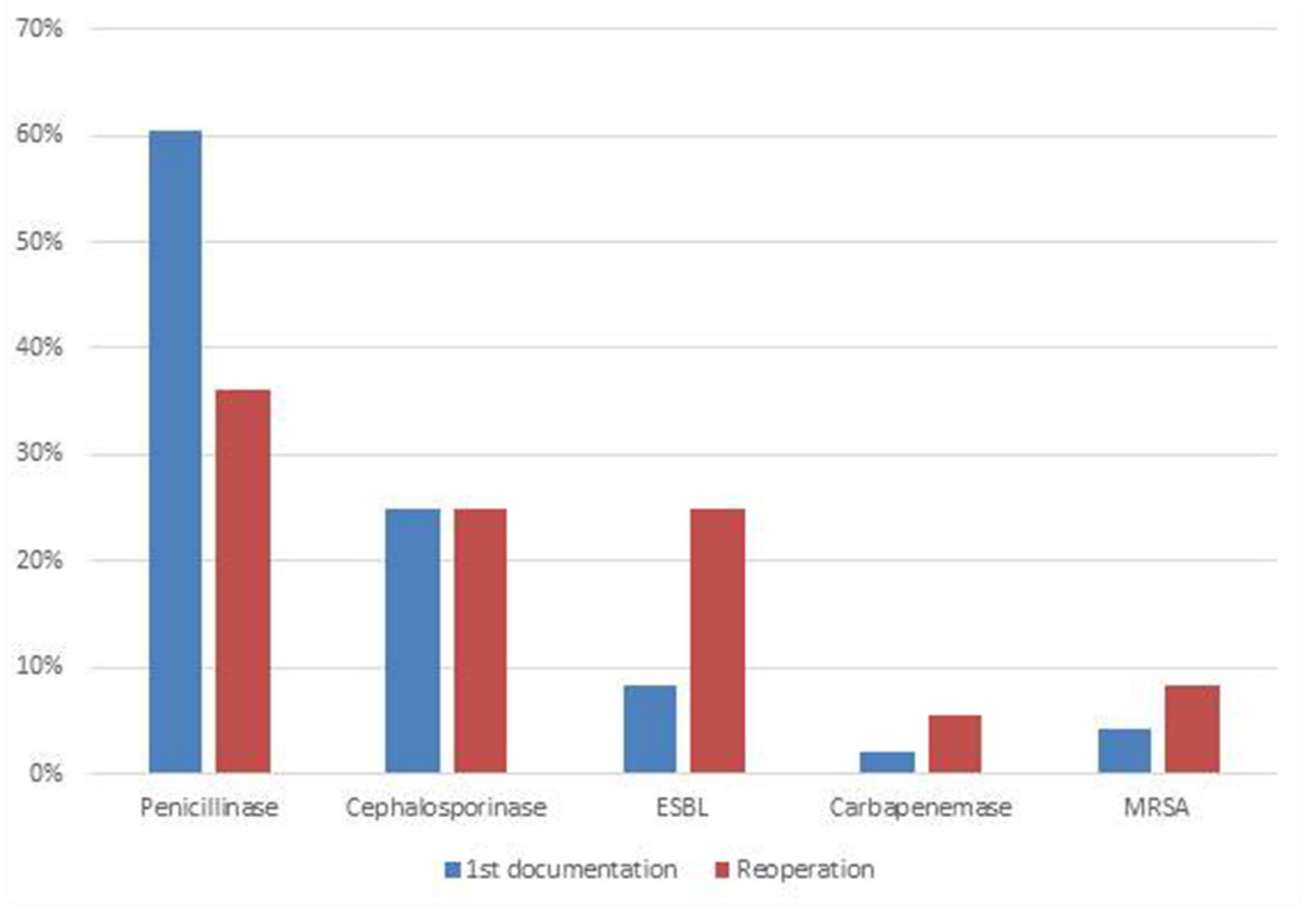
Proportions of identified antibiotic resistances among MDR bacteria during initial surgery and at the time of reoperation. ESBL, extended-spectrum betalactamase; MRSA, methicillin-resistant *Staphylococcus aureus*.

Overall, 65 emerging microorganisms were reported in 30 patients (30%) between the first and second operations. A total of 19 emerging MDR strains were cultured in 15 patients (28%) on the second surgery, involving both Gram-positive and Gram-negative organisms (Table 2, Figure 1). The different mechanisms of resistance are presented in Supplementary Table 3 and Figure 1. Among MDR bacteria, overproduction of intrinsic or plasmid-encoded AmpC cephalosporinases were the most frequent mechanisms of resistance. Cephalosporinase- and ESBL-production were the most frequent emerging mechanisms of resistance.

We expected an increased proportion of emerging MDR during pelvic NSTIs related to the proximity to the bowel flora. However, the emergence of bacteria (*p* = 0.44), persistence of bacteria (*p* = 0.74), and emergence of MDR (*p* = 0.9) were not significantly different in NSTI involving the pelvis area vs. other areas. A comparison of the clinical data of patients with/without the emergence of MDR bacteria is presented in Supplementary Table 4.

The risk factors for emergence of microorganisms at the time of reoperation analyzed in multivariate analysis are presented in Table 3. When considering the variables collected at the time of ICU admission, initial administration of broad-spectrum antibiotic therapy (piperacillin/tazobactam and/or aminoglycosides) was protective factor observed against the emergence of microorganisms on reoperation (Table 3). Concerning the variables collected during the ICU stay, hospital-acquired pneumonia increased significantly the risk of emergence of bacteria.

**Table 3:** Risk factors for emergence of bacteria.

**Variables**	**Univariable analysis**			**Multivariable analysis**			
	**Patients without any emerging microorganisms**	**Patients with emerging microorganisms**	***p*-value**	**Odds ratio**	**2.5% CI**	**97.5% CI**	***p*-value**
**On admission**							
Age median (IQR)	59 (50–68)	56 (50–64)	0.253				
Male gender, *n* (%)	47 (67)	16 (53)	0.258				
BMI, median (IQR)	27 (22–31)	28 (24–36)	0.410				
Anterior SSTI history, *n* (%)	4 (6)	3 (10)	0.430				
Chronic skin disease, *n* (%)	8 (11)	4 (13)	0.749				
Smoker, *n* (%)	43 (61)	11 (37)	0.029	0.35	0.12	0.96	0.04
Anterior antibiotherapy <6 months, *n* (%)	16 (23)	7 (23)	1				
Cardiovascular disease, *n* (%)	27 (40)	7 (23)	0.167				
SOFA score, median (IQR)	5 (3–7)	4 (3–6)	0.175				
SAPS II score, median (IQR)	27 (23–37)	28 (22–38)	0.557				
Haemodynamic failure, *n* (%)	54 (77)	16 (53.3)	0.031	0.26	0.08	0.75	0.01
LRINEC score, median (IQR)	2 (1–4)	2 (1–5)	0.921				
% of corporeal surface, median (IQR)	4.5 (4.5–9)	7 (4.5–17)	0.08	1.02	0.95	1.10	0.6
Adequate empiric antibiotherapy, *n* (%)	56 (80)	23 (77)	0.790				
1st line - aminoglycosides, *n* (%)	21 (30)	3 (10.0)	0.041	0.17	0.03	0.65	0.008
1st line - piperacillin-tazobactam, *n* (%)	29 (41)	4 (13.3)	0.006	0.22	0.05	0.71	0.01
**During ICU stay**							
Antibiotic de-escalation, *n* (%)	41 (62)	16 (57.1)	0.653				
Delay to diagnosis in days, median (IQR)	1 (0–3)	1 (0–2)	0.064	1.00	1.00	1.00	0.07
Total antibiotherapy duration, median (IQR)	14 (10–15)	15 (14–15)	0.476				
Hospital-acquired pneumonia, *n* (%)	3 (4)	8 (27)	0.003	2.04	1.32	3.16	0.005
ICU length of stay, median (IQR)	6 (1–17)	12 (4–35)	0.030	1.00	1.00	1.01	0.37
Delay to reoperation, median (IQR)	3 (1–6)	3 (2–7)	0.47				

We did not observe any link between the clinical criteria collected on ICU admission and emerging MDR bacteria (Table 4). In multivariable analysis, the only identified risk factor for emergence of MDR bacteria on the second surgical procedure was a prolonged delay before initial source control (Table 4).

**Table 4:** Risk factors for emergence of MDR bacteria.

**Variables**	**Univariable analysis**			**Multivariable analysis**			
	**No emergence of MDR strains**	**Emerging MDR strains**	***p*-value**	**Odds ratio**	**2.5% CI**	**97.5% CI**	***p*-value**
**On admission**							
Age median (IQR)	56 (46–67)	62 (56–67)	0.333				
Male gender, *n* (%)	19 (58)	7 (47)	0.543				
BMI, median (IQR)	27 (22–29)	28 (20–43)	0.611				
Anterior SSTI history, *n* (%)	3 (9.1)	2 (13.3)	0.642				
Chronic skin disease, *n* (%)	8 (9)	4 (27)	0.079	2.94	0.68	11.39	0.14
Smoker, *n* (%)	14 (42)	8 (53)	0.543				
Anterior antibiotherapy <6 months, *n* (%)	21 (25)	2 (13)	0.510				
Cardiovascular disease, *n* (%)	29 (35)	5 (33)	1.000				
SOFA score, median (IQR)	5 (3–6)	4 (3–7)	0.849				
SAPS II score, median (IQR)	26 (22–37)	32 (27–37)	0.133				
Haemodynamic failure, *n* (%)	59 (69)	11 (73)	1.000				
LRINEC score, median (IQR)	2 (1–5)	1 (0–2)	0.041	0.84	0.62	1.08	0.19
% of corporeal surface, median (IQR)	4.5 (4.5–9)	9 (4.5–11)	0.201				
Adequate empiric antibiotherapy, *n* (%)	75 (90.4)	16 (94.1)	0.978				
1st line—aminoglycosides, *n* (%)	22 (26)	2 (13)	0.512				
1st line—piperacillin-tazobactam, *n* (%)	29 (34)	4 (27)	0.768				
**During ICU stay**
Antibiotic de-escalation, *n* (%)	51 (64)	6 (43)	0.152				
Delay to diagnosis in days, median (IQR)	0.5 (0–2)	3.5 (1–6)	0.071	1.21	1.02	1.53	0.002
Total antibiotherapy duration, median (IQR)	14 (12–15)	14 (11–15)	0.644				
Hospital-acquired pneumonia, *n* (%)	7 (8)	4 (27)	0.062	9.01	0.39	130.20	0.145
ICU length of stay, median (IQR)	7 (2–19)	8 (6–19)	0.428				
Delay to reoperation, median (IQR)	4 (1–7)	2 (2–3)	0.192				

### Outcomes

The total median duration of antibiotic therapy was 14 (11–15) days. Overall, surgical source control was successful in 90% of the cases. However, 11% of the patients required an amputation, and 13% of them needed a colostomy. A vacuum-assisted closure (VAC) device was used in 30% of the patients, and skin wound repair required skin graft in 24% of patients. Twenty-two patients underwent renal replacement therapy. The median length of ICU and hospital stay were 7 (2–19) and 39 (17–58) days, respectively.

Mortality rates at Day 28, Day 90, and 1 year were 18, 23, and 25%, respectively. Kaplan-Meier estimates of survival among patients [with or without persisting microorganisms (panel A), with or without emerging microorganisms (panel B) and with or without MDR bacteria acquisition (panel C)] are displayed on Supplementary Figure 1. The mortality rate in the entire cohort did not change over the study period (*p* = 0.786, Supplementary Figure 2). The type of microorganisms cultured from the initial surgery did not modify the ICU/hospital length of stay or mortality rate. Persistence of microorganisms was significantly associated with a longer ICU length of stay [17 (7–31) days vs. 5 (1–12) days in patients free of persisting microorganisms, *p* = 0.001], but these persisting microorganisms did not influence the survival rate at 1 year (*p* = 0.135). Similarly, emergence of microorganisms was associated with a longer ICU length of stay [12 (4–35) days vs. 6 (1–18) days in patients free of emerging microorganisms, *p* = 0.052] but was not associated with the survival rate at 1 year (*p* = 0.805). The acquisition of MDR bacteria was not associated with increased ICU or hospital length of stay or survival rate at 1 year.

## Discussion

To the best of our knowledge, this is the first study to focus on the microbiological changes (persistence and emergence of microorganisms) in ICU patients with NSTI who required a reoperation. We observed a shift of Gram-positive to Gram-negative bacteria between the first and the second operation combined with a significant increase in the proportion of MDR bacteria at the time of the second surgery. A broad spectrum first-line antibiotic therapy was associated with reduced emergence of microorganisms. However, adequate antibiotic therapy from the initial surgery did not modify the frequency of emergence of microorganisms or MDR bacteria or the 1-year survival rate of the patients harboring these MDR bacteria.

Our patients are comparable to those described in the most recent epidemiological cohort collected in ICU patients with a significant proportion of NSTI of the limbs and pelvis (30). There is a large variability in terms of the source of NSTI between centers depending on the case mix and the environment. The pelvic site of infection increases the proportions of Gram-negative bacteria among the cultured microorganisms. Most recent and largest studies found between 21 and 34% of NSTI concerning pelvis area (30–32). The proportion of pelvic NSTI in our cohort was slightly more frequent than what was reported in the older literature (33–35). Interestingly, the incidence of Gram-negative isolates in these specific cases was not different from our observations in limb infection, an issue not generalizable to other centers where Gram-positive bacteria are usually the predominant pathogens. The severity scores on admission were quite low in our population despite mechanical ventilation in all the cases and septic shock in half of the patients. Similarly to others, we observed prolonged ICU and hospital stays, which are probably related to the prolonged care required by this specific population (36). The type and susceptibility profiles of the microorganisms cultured from surgical samples and blood cultures on admission are consistent with previous studies with a predominance of Gram-positive isolates in the first operation samples (37–40).

The number of the reoperations was high in our population. Other authors also reported important rates of repeated surgical debridement with a dramatic impact of a delayed initial surgery (>24 h) on mortality (39, 41, 42). Aggressive surgical treatment, especially in patients without favorable evolution, could explain why we did not identify a significant impact of this surgical delay in our cohort. The large proportion of reoperations allowed to make microbiological comparisons. The cultures of the second surgical samples revealed a microbiological shift with decreased proportions of Gram-positive cocci associated with the emergence of Gram-negative bacteria, including *P. aeruginosa*. The microbiological course of NSTI seems to be influenced by the type of EAT. Criteria explaining the persistence of microorganisms were not expected. The observed risk factors for persisting pathogens highlighted various criteria poorly described in the literature, such as the post-operative NSTI, polymicrobial nature of infection or combined hospital acquired pneumonia. These factors could be easily detected and might be useful for detection of patients at risk of reoperation.

A first-line broad-spectrum antibiotic therapy seems to play a protective role in the emergence of microorganisms between the two surgeries. This unexpected observation is in contradiction with the current trend of limiting antibiotic selection pressure. However, the 2018 WSES/SIS-E consensus conference stressed the need for aggressive broad-spectrum EAT to target Gram-positive, Gram-negative and anaerobic bacteria (2). Paradoxically, adequacy of antibiotic treatment did not change the frequency of emergence of microorganisms regardless of the period analyzed or the outcomes of the patients. Concomitantly, we observed a progressive emergence of MDR strains over time. These findings are consistent with the results found in other ICU surgical populations, such as patients treated for intra-abdominal infections in whom an emergence of MDR bacteria was gradually observed with the number of reoperations (19). According to our data, the use of broad-spectrum antibiotic therapies, such as piperacillin-tazobactam and aminoglycoside or carbapenems, did not seem to influence the emergence of MDR bacteria. Only delayed diagnosis and antibiotic therapy appear to be independent factors associated with the emergence of MDR bacteria. This finding could reinforce the need for early and aggressive management from the initial phase of anti-infective therapy.

We observed some variabilities in terms of emerging bacteria between the different location of NSTI, which are potentially related to inhomogeneous diffusion of antibiotics or local microbiota issues. The quality of source control and its delay can also be variable (43). Persistence and emergence of microorganisms were not significantly associated with survival rates but were associated with prolonged ICU length of stay. Interestingly, emerging MDR bacteria were frequently reported in our cohort without significant changes in survival rates or ICU/hospital length of stay.

Our work has some limitations. First, it is a retrospective, monocentric and observational study with a relatively small sample conferring a low level of evidence. We consider this study to be strictly exploratory. Thus, it is not possible to draw definite conclusions from such a limited number of cases and our observations need to be confirmed in a larger multicentric approach. This comment is relevant for the analysis of the relationship between antibiotic selection pressure and emergence of MDR microorganism which needs a specific investigation. Second, the long period of inclusion, which was necessary regarding the low incidence of NSTI in ICU patients, is questionable. However, we observed that the 1-year mortality was stable throughout the inclusion period among patients with NSTI. In addition, we cannot exclude that confounding factors for mortality may not have been included, mitigating the multivariate analysis results. Third, the surgical samples were examined only by culture-based techniques, which have some limitations. All the pathogens involved in NSTI could be not detected, especially if antibiotic therapy was initiated before sampling (44), or in case of hardly or uncultivable microorganisms like anaerobes. Culture-free techniques (16S rRNA amplicon and metagenomics shotgun sequencing) could have provided a deeper insight into the global taxonomic and resistance profiles of the microbial population. Indeed, these techniques are highly effective in characterizing microbial population and very complementary to culture-based techniques (45). In addition, persistence of the microorganisms was assessed according to a phenotypic description without any genotypic confirmation. Another point to consider is the impossible differentiation between wound colonization and authentic infection. Indeed, focusing on bacterial species *per se* may cause inclusion of irrelevant cases and thereby over-interpretation of observations. The only clinical surrogates of the pathogenic role of the microorganisms isolated on the second surgery are the persistent organ dysfunctions and high white blood cell counts. This issue prompts cautious consideration of these results and the need for antibiotic escalation in case of abatement of clinical signs of infection. The emergence of MDR bacteria was only assessed for the surgical wound. However, a broader analysis of the emergence of MDR bacteria could also be of interest, especially in terms of digestive carriage. Finally, the pharmacokinetic characteristics of anti-infective agents and their tissue diffusion could play a role in the mechanisms of persistence or emergence of microorganisms, but this issue was not monitored on a routine basis, representing a limitation in the interpretation of the results. However, the pharmacokinetic characteristics of antibiotics have rarely been reported in the management of NSTI.

The concept of emergence and persistence of microorganisms encourages us to take a closer look to the issue of colonization, which is similar to the practice used in burned patients. A combination of close clinical evaluation, repeated biomarkers and microbiological mapping during the first days of management of NSTIs could be interesting to evaluate the decreased duration of anti-infective therapy and consequently antibiotic selection pressure (34, 35).

## Conclusion

In our cohort of ICU patients admitted for NSTI, we highlighted the high frequency of persisting and emerging microorganisms and their important consequences in terms of morbi-mortality with a prolonged ICU length of stay. Broad-spectrum first-line therapy was associated with a decreased emergence of microorganisms. We showed a shift of Gram-positive to Gram-negative bacteria between the first and the second operation combined with a significant increase in the proportion of MDR bacteria at the time of the second surgery. However, use of an adequate antibiotic therapy from the initial surgery did not modify the frequency of emergence of microorganisms or MDR bacteria or the 1-year survival rate of the patients facing these MDR bacteria.

## Data Availability Statement

The raw data supporting the conclusions of this article will be made available by the authors, without undue reservation.

## Ethics Statement

The studies involving human participants were reviewed and approved by a French Institutional Review Board (Comité d'Éthique de la Recherche en Anesthésie-Réanimation, IRB number 00010254-2020-153). Written informed consent for participation was not required for this study in accordance with the national legislation and the institutional requirements.

## Author Contributions

MT participated in study design, acquisition of data, analysis and interpretation of data, performed the statistical analysis, and drafted the manuscript. ST and PM participated in study design, acquisition of data, analysis and interpretation of data, and drafted the manuscript. AT-D, LR, BL-J, JD, NZ, MB-R, PT, AS, and EA were involved in the analysis and interpretation of data. NG participated in the design of the study, were involved in the analysis and interpretation of data and drafted the manuscript. All authors have read and approved the manuscript.

## Conflict of Interest

The authors declare that the research was conducted in the absence of any commercial or financial relationships that could be construed as a potential conflict of interest. The reviewer, DG, declared a past co-authorship with two of the authors (EA and PM) at time of review to the handling editor.

## Footnotes

^1^EUCAST: Clinical breakpoints and dosing of antibiotics. Available online at: https://eucast.org/clinical_breakpoints/ (accessed June 22, 2020).
