# Supplementary material for: Dynamic Changes in Microbial Composition During Necrotizing Soft-Tissue Infections in ICU Patients
Source: Front Med (Lausanne). 2021 Mar 4;7:609497. doi: 10.3389/fmed.2020.609497 (PMC7969649; doi:10.3389/fmed.2020.609497)
Supplement: Supplementary file 1 [file Data_Sheet_1.PDF]

Figure S1: Kaplan-Meier estimates of survival among patients with or without persistence of microorganism (panel A), with or without emerging microorganism (panel B) and with or without emerging MDR microorganisms (panel C).

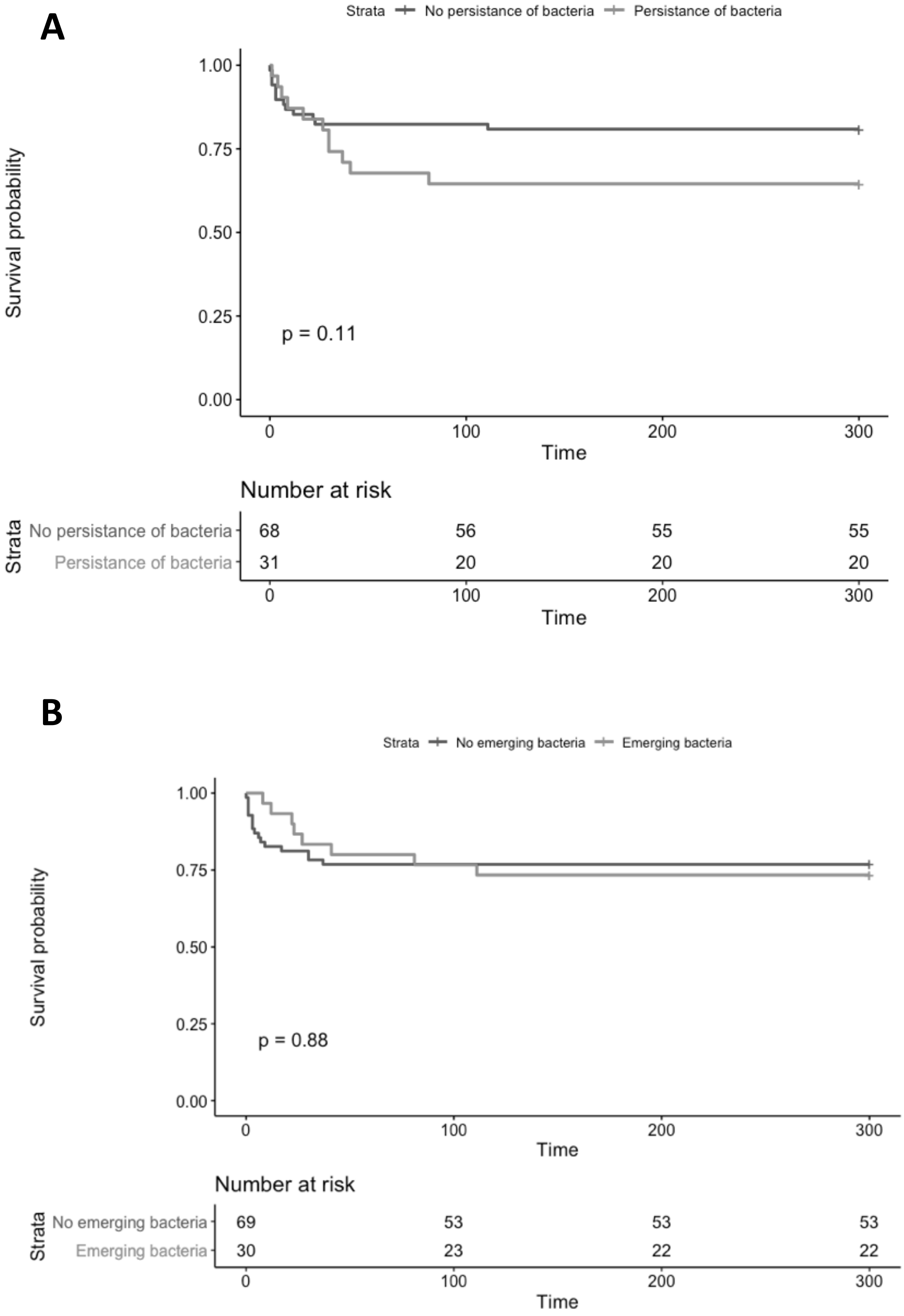

C

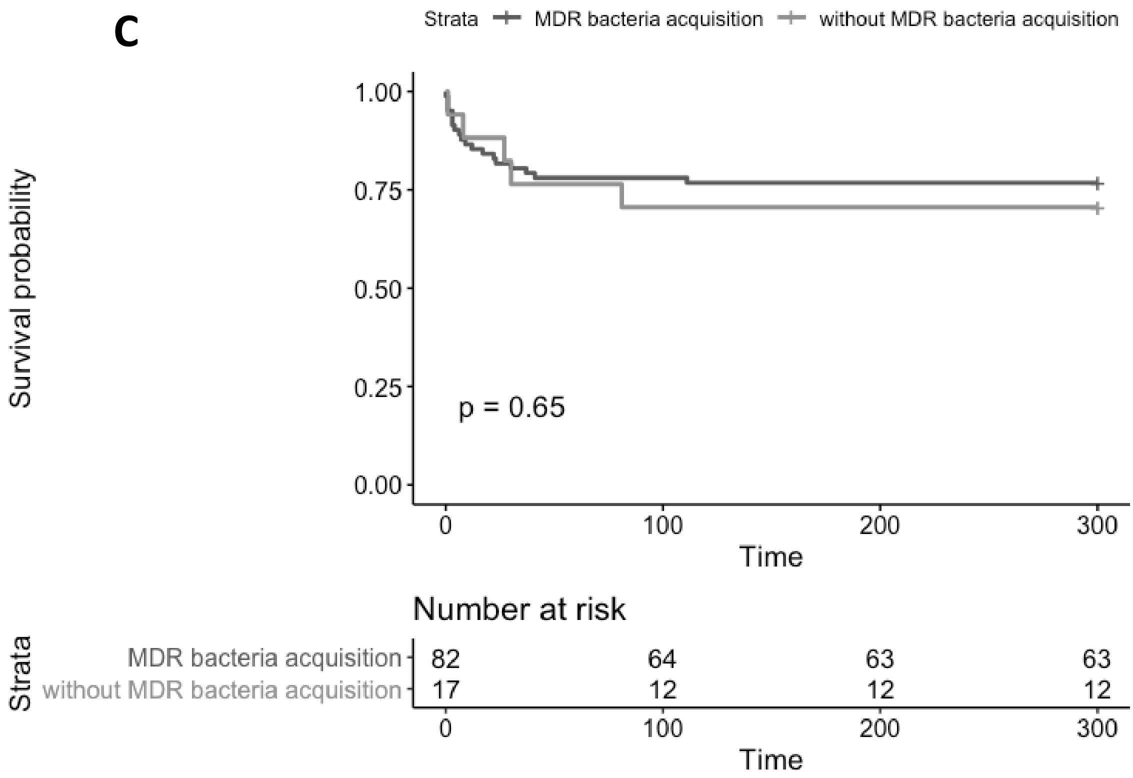

Strata
MDR bacteria acquisition
without MDR bacteria acquisition
Survival probability
1.00
0.75
0.50
0.25
0.00
p = 0.65
0
100
200
300
Time
Number at risk
Strata
MDR bacteria acquisition
without MDR bacteria acquisition
82
64
63
63
17
12
12
12
0
100
200
300
Time
