# Supplementary material for: Dynamic Changes in Microbial Composition During Necrotizing Soft-Tissue Infections in ICU Patients
Source: Front Med (Lausanne). 2021 Mar 4;7:609497. doi: 10.3389/fmed.2020.609497 (PMC7969649; doi:10.3389/fmed.2020.609497)
Supplement: Supplementary file 2 [file Data_Sheet_2.PDF]

Figure S2: Mortality over the years in the entire cohort

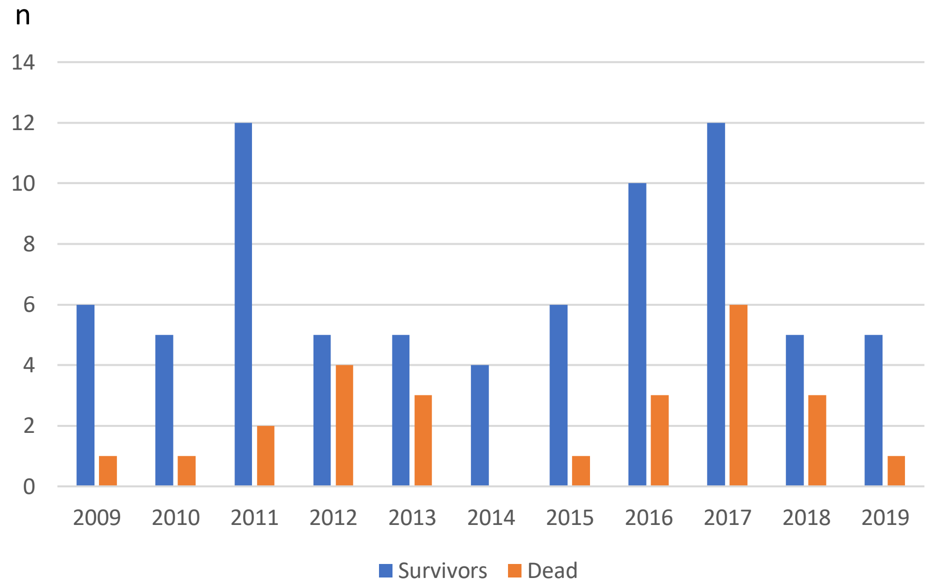

Non-parametric Kruskal-Wallis Rank Sum Test over the years is not significant ($p=0.786$).
