# Supplementary material for: Dynamic Changes in Microbial Composition During Necrotizing Soft-Tissue Infections in ICU Patients
Source: Front Med (Lausanne). 2021 Mar 4;7:609497. doi: 10.3389/fmed.2020.609497 (PMC7969649; doi:10.3389/fmed.2020.609497)
Supplement: Supplementary file 3 [file Data_Sheet_3.PDF]

Table S1: Comparison of different patients' microbe NSTI documentation combinations

	**Mono microbial**	**Poly microbial**	**Poly microbial ≥3**	**Gram-negative bacteria**	*E. coli*	*Klebsiella spp.*	*Enterobacter spp.*	Non-fermenting Gram-negative bacilli	**Gram-positive bacteria**	*Staphylococcus aureus*	Coagulase-negative staphylococci	Strepto-cocci	Entero-cocci	**Anae-robes**	**Fungi**
**Gram-negative bacteria**	11	17	10	5	2	1	0	2	8	0	1	3	4	3	1
Escherichia coli	5	7	5	2	0	1	0	1	3	0	0	1	2	2	0
Klebsiella spp.	1	4	2	1	1	0	0	0	3	0	0	1	2	0	0
Enterobacter spp.	0	2	1	0	0	0	0	0	1	0	1	0	0	0	1
Non-fermenting Gram-negative bacilli	2	4	2	2	1	0	0	1	1	0	0	1	0	1	0
**Gram-positive bacteria**	32	28	12	8	3	3	1	1	16	1	4	10	1	3	1
Staphylococcus aureus	9	2	0	0	0	0	0	0	1	0	1	0	0	1	0
Coagulase-negative staphylococci	2	5	1	1	0	0	1	0	4	1	0	3	0	0	0
Streptococci	11	16	8	3	1	1	0	1	10	0	3	6	1	2	1
Enterococci	2	5	3	4	2	2	0	0	1	0	0	1	0	0	0
**Anaerobes**	2	8	8	3	2	0	0	1	3	1	0	2	0	2	0
**Fungi**	0	2	2	1	0	0	1	0	1	0	0	1	0	0	0
**Total**	45	55	32	17	7	4	2	4	28	2	5	16	5	8	2
