# Supplementary material for: Dynamic Changes in Microbial Composition During Necrotizing Soft-Tissue Infections in ICU Patients
Source: Front Med (Lausanne). 2021 Mar 4;7:609497. doi: 10.3389/fmed.2020.609497 (PMC7969649; doi:10.3389/fmed.2020.609497)
Supplement: Supplementary file 4 [file Data_Sheet_4.PDF]

Table S2: Microbiological results and evolution between the first surgery and reoperation depending on the location of NSTI.

	**Limbs**		**Pelvis**		**Cephalic**		**Trunk**	
n (%)	**52**		**28**		**23**		**13**	
	1st surgery	Reoperation	1st surgery	Reoperation	1st surgery	Reoperation	1st surgery	Reoperation
Aerobes, n (%)	**69 (88)**	**37 (88)**	**38 (78)**	**25 (86)**	**35 (88)**	**28 (90)**	**21 (75)**	**16 (76)**
Gram positive, n (%)	38 (49)	16 (38)	18 (37)	10 (34)	26 (65)	14 (45)	12 (43)	6 (29)
Enterococci, n (%)	4 (5)	3 (7)	5 (10)	3 (10)	3 (8)	1 (3)	5 (18)	2 (10)
Streptococci, n (%)	21 (27)	5 (12)	9 (18)	1 (3)	15 (38)	6 (19)	5 (18)	2 (10)
Staphylococci, n (%)	13 (17)	8 (19)	4 (8)	6 (21)	8 (20)	7 (23)	2 (7)	2 (10)
Staphylococcus aureus, n (%)	10 (13)	3 (7)	2 (4)	1 (3)	3 (8)	4 (13)	2 (7)	1 (5)
Coagulase-negative staphylococci, n (%)	3 (4)	5 (12)	2 (4)	5 (17)	5 (13)	3 (10)	0 (0)	1 (5)
Gram negative, n (%)	31 (40)	21 (50)	20 (41)	15 (52)	9 (23)	14 (45)	9 (32)	10 (48)
Enterobacteriaceae, n (%)	25 (7)	15 (36)	14 (29)	10 (34)	8 (20)	8 (26)	8 (29)	7 (33)
Escherichia coli, n (%)	8 (3)	9 (21)	10 (20)	8 (28)	3 (8)	3 (10)	6 (21)	3 (14)
Enterobacter spp., n (%)	1 (1)	3 (7)	1 (2)	1 (3)	0 (0)	1 (3)	0 (0)	3 (14)
Klebsiella spp., n (%)	8 (10)	5 (12)	0 (0)	3 (10)	2 (5)	5 (16)	1 (4)	4 (19)
Non-fermenting Gram-negative bacilli, n (%)	6 (8)	6 (14)	6 (12)	5 (17)	1 (3)	6 (19)	1 (4)	3 (14)
Anaerobes, n (%)	**8 (10)**	**3 (7)**	**10 (20)**	**2 (7)**	**3 (8)**	**1 (3)**	**5 (18)**	**2 (10)**
Bacteroides spp., n (%)	4 (5)	3 (7)	6 (12)	2 (7)	1 (3)	0 (0)	1 (4)	2 (10)
Fungi, n (%)	**1 (1)**	**2 (5)**	**1 (2)**	**2 (7)**	**2 (5)**	**2 (6)**	**2 (7)**	**3 (14)**
Total number of microorganisms, n (%)	**78 (100)**	**42 (100)**	**49 (100)**	**29 (100)**	**40 (100)**	**31 (100)**	**28 (100)**	**21 (100)**
Total number of MDR bacteria, n (%)	**4 (100)**	**9 (100)**	**4 (100)**	**4 (100)**	**2 (100)**	**8 (100)**	**0 (100)**	**3 (100)**
MRSA, n (%)	2 (50)	2 (22)	0 (0)	0 (0)	0 (0)	1 (13)	0 (0)	1 (33)
ESBL, n (%)	1 (25)	3 (33)	3 (75)	2 (50)	0 (0)	4 (50)	0 (0)	1 (33)
Derepressed AmpC betalactamase, n (%)	0 (0)	1 (11)	0 (0)	0 (0)	2 (100)	2 (25)	0 (0)	0 (0)
Carbapenemase, n (%)	1 (25)	1 (11)	1 (25)	1 (25)	0 (0)	0 (0)	0 (0)	1 (33)
XDR Pseudomonas, n (%)	0 (0)	2 (22)	0 (0)	1 (25)	0 (0)	1 (13)	0 (0)	0 (0)
