# Supplementary material for: Dynamic Changes in Microbial Composition During Necrotizing Soft-Tissue Infections in ICU Patients
Source: Front Med (Lausanne). 2021 Mar 4;7:609497. doi: 10.3389/fmed.2020.609497 (PMC7969649; doi:10.3389/fmed.2020.609497)
Supplement: Supplementary file 5 [file Data_Sheet_5.PDF]

Table S3: Proportions of identified antibiotic resistances during initial surgery (174 strains) and at the time of reoperation (95 strains). ESBL: Extended-spectrum betalactamase; MRSA: Methicillin-resistant *Staphylococcus aureus.*

	**1st documentation**	**Reoperation**
Penicillinase	29	13
Cephalosporinase	12	9
ESBL	4	9
Carbapenemase	1	2
MRSA	2	3
