# Supplementary material for: Dynamic Changes in Microbial Composition During Necrotizing Soft-Tissue Infections in ICU Patients
Source: Front Med (Lausanne). 2021 Mar 4;7:609497. doi: 10.3389/fmed.2020.609497 (PMC7969649; doi:10.3389/fmed.2020.609497)
Supplement: Supplementary file 6 [file Data_Sheet_6.PDF]

Table S4: Comparison of the characteristics and outcomes between patients with/without emergence of MDR bacteria.

Variables	No emergence of MDR strains (n=85)	Emerging MDR strains (n=15)	p
**Demographic data**			
Age (years), median [IQR]	59 [50-68]	56 [52-63.50]	0.519
Male gender, n (%)	56 (66)	7 (47)	0.245
Weight (kg), median [IQR]	82 [68-94]	85 [52-105]	0.59
BMI (kg/m^2), median [IQR]	28 [23-32]	30 [18-35]	0.667
**Underlying diseases**			
Chronic obstructive pulmonary disease, n (%)	13 (15)	2 (13)	1
Cardiovascular disease, n (%)	21 (25)	4 (27)	1
Diabetes mellitus, type I or II, n (%)	22 (26)	2 (13)	0.512
Peripheral vascular disease, n (%)	16 (19)	1 (7)	0.456
Immunosuppression, n (%)	9 (11)	1 (7)	1
Cancer, n (%)	19 (22)	4 (27)	0.743
Active smoking	45 (53)	9 (60)	0.780
Alcohol use, n (%)	15 (18)	5 (33)	0.173
Use of steroids or NSAI drugs, n (%)	7 (44)	0 (0)	1.000
Charlson score median [IQR]	3 [2-6]	5 [2-5]	0.936
**CT scan on the first examination, n (%)**			
Median delay from first exam to CT scan, days [IQR]	1 [0-5]	2 [0.5-3]	0.839
Deep abscess, n (%)	26 (31)	3 (20)	0.542
Bullae, n (%)	17 (20)	1 (7)	0.293
Fasciitis signs, n (%)	14 (17)	3 (20)	0.718
Osteitis signs, n (%)	7 (8)	2 (13)	0.622
**Severity criteria**			
SAPS II score on admission, median [IQR]	26 [22-37]	32 [27-37]	0.106
SOFA score on admission, median [IQR]	5 [3-6]	4 [3-9]	0.785
LRINEC score on admission, median [IQR]	2 [1-5]	2 [0-4]	0.18
**Treatment**			
Vaso-active support on admission, n (%)	42 (51)	11 (73)	0.159
Renal replacement therapy, n (%)	15 (19)	7 (47)	0.039
Length of mechanical ventilation, median [IQR]	3 [0-12]	3 [0.5-17]	0.593
Antibiotic duration, median [IQR]	14 [12-15]	14 [11-15]	0.6
Vacuum-assisted closure device, n (%)	23 (27)	5 (33)	0.756
Skin graft, n (%)	20 (24)	4 (27)	0.752
Amputation, n (%)	14 (17)	1 (7)	0.453
**Outcomes**			
Length of hospital stay in days, median [IQR]	40 [18-60]	43 [31-53]	0.814
Length of ICU stay in days, median [IQR]	7 [2-19]	9 [6-29]	0.202
Death, n (%)	20 (24)	5 (33)	0.518
